# Factors That Might Affect SARS-CoV-2 Transmission Among Foreign-Born and U.S.-Born Poultry Facility Workers — Maryland, May 2020

**DOI:** 10.15585/mmwr.mm6950a5

**Published:** 2020-12-18

**Authors:** Beth L. Rubenstein, Stefanie Campbell, Alysha R. Meyers, David A. Crum, Clifford S. Mitchell, Jeré Hutson, D’Ann L. Williams, Schabbethai S. Senesie, Zunera Gilani, Steven Reynolds, Bianca Alba, Stephanie Tavitian, Kristian Billings, Lina Saintus, Stephen B. Martin, Hugh Mainzer

**Affiliations:** ^1^CDC COVID-19 Response Team; ^2^Epidemic Intelligence Service, CDC; ^3^Maryland Department of Health.

Numerous recent assessments indicate that meat and poultry processing facility workers are at increased risk for infection with SARS-CoV-2, the virus that causes coronavirus disease 2019 (COVID-19) (*1*–*4*). Physical proximity to other workers and shared equipment can facilitate disease transmission in these settings (*2*–*4*). The disproportionate number of foreign-born workers employed in meat and poultry processing reflects structural, social, and economic inequities that likely contribute to an increased COVID-19 incidence in this population* (*5*). In May 2020, the Maryland Department of Health and CDC investigated factors that might affect person-to-person SARS-CoV-2 transmission among persons who worked at two poultry processing facilities.^†^ A survey administered to 359 workers identified differences in risk factors for SARS-CoV-2 infection between workers born outside the United States and U.S.-born workers. Compared with U.S.-born workers, foreign-born workers had higher odds of working in fixed locations on the production floor (odds ratio [OR] for cutup and packaging jobs = 4.8), of having shared commutes (OR = 1.9), and of living with other poultry workers (OR = 6.0). They had lower odds of participating in social gatherings (OR for visits to family = 0.2; OR for visits to friends = 0.4), and they visited fewer businesses in the week before the survey than did their U.S.-born coworkers. Some workplace risk factors can be mitigated through engineering and administrative controls focused on the production floor, and this will be of particular benefit to the foreign-born workers concentrated in these areas. Employers and health departments can also partner with local organizations to disseminate culturally and linguistically tailored messages about risk reduction behaviors in community settings, including shared transportation^§^ and household members dwelling in close quarters.^¶^

During a 2-day period in May 2020, interviews were conducted with a convenience sample of on-duty workers selected by management from two poultry processing facilities during the morning and evening shifts. Management selected workers assigned to different areas of the facility to minimize disruptions to production. Interviews were guided by a structured questionnaire that collected information about workers’ demographic characteristics (e.g., sex, age, and country of birth) and their risks for contracting SARS-CoV-2 during the week preceding the interview (e.g., commuting, large household size, presence of other poultry workers in the household, visits to businesses, gatherings with friends and family, and COVID-19 information sources). Foreign-born workers were defined as workers born outside the United States, including immigrants and refugees. The questionnaire was developed in English and translated into Haitian Creole and Spanish. Interview data were combined with employment records provided by both facilities on workers’ race and ethnicity, assigned roles, shifts, and years of employment. Roles were categorized to correspond with work locations. Fixed jobs on the production floor (e.g., cutup and packaging, evisceration, and receiving) were considered high-risk because they involve physical proximity to other workers and have been associated with SARS-CoV-2 transmission in other meat processing facilities (*2*–*4*). Cold temperature work areas were also considered high-risk because cold could prolong virus stability and facilitate transmission (*6*). Fixed jobs were compared with jobs that involved multiple work areas because the latter tend to be managerial or maintenance positions with more flexibility to maintain physical distance and have less contact with high-touch surfaces. Data were analyzed descriptively, and crude ORs were calculated to analyze the strength of the associations between being foreign-born and selected characteristics. For continuous variables, comparisons were based on the Wilcoxon rank sum test. Both structural factors (i.e., characteristics reflecting economic, social, policy, and organizational environments, such as work areas, housing and transportation) and behavioral factors (i.e., individual-level actions and practices, such as visits to businesses, social gatherings and use of masks) were evaluated. SAS software (version 9.4; SAS Institute) was used for all analyses. All activities were reviewed by CDC and were conducted consistent with applicable federal law and CDC policy.** 

Among 2,345 total workers in facilities A and B, 359 (14.7%) were interviewed, including 154 (42.9%) from facility A (24.4% of facility A workers) and 205 (57.1%) from facility B (11.4% of facility B workers) (Table). The sample was evenly distributed by sex (48.7% female, 171); median age was 41.1 years (interquartile range = 29.7–53.7 years). Non-Hispanic Black or African American persons accounted for 241 (73.3%) workers, non-Hispanic White persons for 54 (16.4%), and Hispanic or Latino persons for 21 (6.4%). Overall, 135 (37.8%) interviewed workers were foreign-born, 89 (65.9%) of whom were from Haiti.

**TABLE:** Characteristics and activities of poultry processing workers, overall and by country of birth, and crude odds ratios (ORs) for being foreign-born — Maryland, May 2020

Characteristic or activity*	No. (column %)			Crude OR (95% CI) (categorical); p-value (continuous)^†^
	All (N = 359)	Country of birth		
		Foreign-born (n = 135)	U.S.-born (n = 224)	
**Demographics**				
**Categorical variables**				
**Female**	171 (48.7)	70 (53.9)	101 (45.7)	1.4 (0.9–2.2)
**Race and ethnicity** ^§^				
Black	241 (73.3)	89 (80.2)	152 (69.7)	10.0 (3.0–32.8)
White^¶^	54 (16.4)	3 (2.7)	51 (23.4)	Referent
Hispanic/Latino	21 (6.4)	13 (11.7)	8 (3.7)	27.6 (6.4–118.9)
Asian	6 (1.8)	5 (4.5)	1 (0.5)	—^†^
Other race	7 (2.1)	1 (0.9)	6 (2.8)	—^†^
**Interview language**				
English^¶^	243 (67.7)	23 (17.0)	220 (98.2)	Referent
Haitian Creole	79 (22.0)	79 (58.5)	0 (0.0)	Undefined
Spanish	37 (10.3)	33 (24.4)	4 (1.8)	78.9 (25.7–242.6)
**Continuous variables**				
**Median age in years (IQR)**	41.1 (29.7–53.7)	39.4 (30.7–51.2)	42.6 (29.1–54.2)	0.46
Structural factors**				
**Categorical variables**				
**Facility**				
Facility A	154 (42.9)	41 (30.4)	113 (50.5)	0.4 (0.3–0.7)
Facility B^¶^	205 (57.1)	94 (69.6)	111 (49.6)	Referent
**Shift** ^††^				
Morning shift^¶^	178 (54.3)	42 (38.2)	136 (62.4)	Referent
Evening shift	150 (45.7)	68 (61.8)	82 (37.6)	2.7 (1.7–4.3)
**Area of plant**				
Cutup and packaging	198 (56.6)	96 (73.3)	102 (46.6)	4.8 (2.3–10.0)
Evisceration	49 (14.0)	20 (15.3)	29 (13.2)	3.5 (1.5–8.5)
Multiple areas^¶^	61 (17.4)	10 (7.6)	51 (23.3)	Referent
Offsite^§§^	3 (0.9)	0 (0.0)	3 (1.4)	Undefined
Outside the production floor	13 (3.7)	0 (0.0)	13 (5.9)	Undefined
Receiving/Live hang/Scald and pick	13 (3.7)	3 (2.3)	10 (4.6)	1.5 (0.4–6.6)
Shipping/Cooler	13 (3.7)	2 (1.5)	11 (5.0)	0.9 (0.2–4.8)
**Temperature of work area** ^¶¶^				
Cold	211 (60.3)	98 (72.6)	113 (50.5)	4.4 (2.1–9.2)
Hot	62 (17.7)	23 (17.0)	39 (17.4)	3.0 (1.3–7.0)
Multiple areas^¶^	61 (17.4)	10 (7.4)	51 (22.8)	Referent
Other	16 (4.6)	0 (0.0)	16 (7.1)	Undefined
Commute pattern***				
Alone	190 (52.9)	55 (40.7)	135 (60.3)	0.5 (0.3–0.7)
Shared, with other household members	52 (14.5)	23 (17.0)	29 (13.0)	1.4 (0.8–2.5)
Shared, with persons from outside the household	128 (35.7)	61 (45.2)	67 (29.9)	1.9 (1.2–3.0)
**At least one other person in the household currently works at a poultry plant**	137 (38.2)	86 (63.7)	51 (22.8)	6.0 (3.7–9.5)
Source of information about COVID-19***				
Church	3 (0.8)	3 (2.2)	0 (0.0)	Undefined
Health officials	13 (3.6)	3 (2.2)	10 (4.5)	—^†^
Internet	121 (33.7)	33 (24.4)	88 (39.3)	0.5 (0.3–0.8)
Newspaper	7 (2.0)	4 (3.0)	3 (1.3)	—^†^
Person-to-person	56 (15.6)	23 (17.0)	33 (14.7)	1.2 (0.7–2.1)
Radio	24 (6.7)	17 (12.6)	7 (3.1)	4.5 (1.8–11.1)
Social media	66 (18.4)	25 (18.5)	41 (18.3)	1.0 (0.6–1.8)
TV news	257 (71.6)	90 (66.7)	167 (74.6)	0.7 (0.4–1.1)
Work	110 (30.6)	39 (28.9)	71 (31.7)	0.9 (0.5–1.4)
**Continuous variables**				
**Median number of persons in the household (IQR)**	4.0 (2.0–5.0)	4.0 (3.0–5.0)	3.0 (2.0–4.0)	<0.001
**Behavioral factors** ^†††^				
**Categorical variables**				
**Wears a mask during shared commute** ^§§§^	104 (81.9)	57 (93.4)	47 (71.2)	5.8 (1.8–18.1)
Business visits in the past week***				
Beauty salon or barbershop	10 (2.8)	2 (1.5)	8 (3.6)	—^†^
Gas station	188 (52.4)	52 (38.5)	136 (60.7)	0.4 (0.3–0.6)
Grocery store	265 (73.8)	94 (69.6)	171 (76.3)	0.7 (0.4–1.1)
Laundromat	70 (19.5)	36 (26.7)	34 (15.2)	2.0 (1.2–3.4)
Liquor store	51 (14.2)	9 (6.7)	42 (18.8)	0.3 (0.1–0.7)
Medical office/Clinic/Hospital	26 (7.2)	11 (8.2)	15 (6.7)	1.2 (0.6–2.8)
Post office	24 (6.7)	5 (3.7)	19 (8.5)	0.4 (0.2–1.1)
Restaurant or bar	25 (7.0)	3 (2.2)	22 (9.8)	—^†^
Other store	26 (7.2)	9 (6.7)	17 (7.6)	0.9 (0.4–2.0)
Household visits in the past week***				
Received visitors at own home	110 (30.9)	25 (18.8)	85 (38.1)	0.4 (0.2–0.6)
Went to family member's home	77 (21.5)	12 (8.9)	65 (29.0)	0.2 (0.1–0.5)
Went to friend's home	36 (10.0)	8 (5.9)	28 (12.5)	0.4 (0.2–1.0)
**Continuous variables**				
**Median number of places visited in the past week (IQR)** ^¶¶¶^	1.0 (1.0–2.0)	1.0 (1.0–2.0)	2.0 (1.0–3.0)	<0.01

**Abbreviations:** CI = confidence interval; COVID-19 = coronavirus disease 2019; IQR = interquartile range.

* Some workers were missing data on sex (eight), age (two), race and ethnicity (30), shift (30), area of plant (nine), and temperature of work area (nine).

^†^ For categorical variables: ORs and 95% CIs of foreign-born workers compared with U.S.-born workers. ORs were only calculated for categories with at least five workers in each cell. For continuous variables: p-values for Wilcoxon rank sum test for foreign-born workers compared with U.S.-born workers.

^§^ Employment records combined race and ethnicity into a single variable and might have underestimated the Hispanic/Latino population.

^¶^ Reference group for ORs.

** Structural factors are characteristics or activities reflecting economic, social, policy, and organizational environments.

^††^ One respondent who worked the third shift (overnight) was excluded.

^§§^ Off-site refers to positions that are not located in the processing building, including delivery, wastewater, and human resource operations.

^¶¶^ Certain areas of the production floor are kept at specific temperatures to facilitate production. For example, areas where carcasses are scalded and defeathered are hot, and areas where carcasses are chilled are cold. Office areas are kept at room temperature.

*** Multiple answers were permitted, and each answer choice was analyzed as the odds of answering “yes” for that option compared with the odds of answering “no” (“no” was the reference group).

^†††^ Behavioral factors are characteristics or activities reflecting individual-level actions and practices.

^§§§^ Percentage who wore masks was calculated out of 127 workers who commuted to work with persons outside their household (one shared commuter was missing data for this question).

^¶¶¶^ Sum of business and household visits in the past week.

Among all interviewed workers, 128 (35.7%) commuted to work via shared transport with persons from outside their household; among these, 104 (81.9%) reported wearing masks during transit.^††^ During the week before the interview, 265 (73.8%) interviewees visited grocery stores, and 188 (52.4%) visited gas stations. Visits to other businesses (e.g., restaurants, bars, and hair salons) were uncommon. Some workers participated in social gatherings: 77 (21.5%) visited family members, 36 (10.0%) visited friends, and 110 (30.9%) hosted a visitor in their home.

The profile of foreign-born workers differed from that of U.S.-born workers in several ways. Compared with U.S.-born workers, foreign-born workers were disproportionately concentrated in certain jobs and areas of the facility (OR for workers assigned to cutup and packaging jobs versus those assigned to multiple areas = 4.8; OR for those assigned to cold-temperature versus to multiple-temperature work areas = 4.4).^§§^ The odds of foreign-born workers commuting with persons from outside their household were 1.9 times the odds for U.S.-born workers. The median size of foreign-born workers’ households was four persons, and that of U.S.-born workers was three (p<0.01). The odds of foreign-born workers living with other poultry workers were 6.0 times that of U.S.-born workers. Foreign-born workers were less likely than were U.S.-born workers to get information about COVID-19 from the Internet (OR = 0.5) and more likely to get information from radio (OR = 4.5).

In the week before being interviewed, foreign-born workers were less likely than were U.S.-born workers to have visited most businesses, including gas stations (OR = 0.4) and liquor stores (OR = 0.3), to have visited a family member’s home (OR = 0.2) or a friend’s home (OR = 0.4), or to have received visitors in their own home (OR = 0.4). Foreign-born workers had higher odds of wearing a mask during shared commutes, compared with U.S.-born workers who also had shared commutes (OR = 5.6).

## Discussion

In a sample of poultry processing workers in two Maryland facilities, all workers reported risks that might affect SARS-CoV-2 transmission. Structural factors were more apparent than were behavioral factors, especially among foreign-born workers. Some structural factors (e.g., shared transportation and larger household size) are common features of foreign-born populations in the United States (*7*,*8*). However, other structural factors are more specific to the workplace and can be mitigated through engineering and administrative controls. For example, in other meat processing facilities, workers with fixed jobs on the production floor had the highest SARS-CoV-2 attack rates and the most frequent contact with ill coworkers^¶¶^ (*2*–*4*). Engineering and administrative controls (e.g., modified alignment of workstations along processing lines, adequate ventilation, installation of physical barriers and handwashing stations, staggering of arrival and break times, and visual cues about social distancing) might reduce risk for SARS-CoV-2 transmission for all workers on the production floor, many of whom are foreign-born (*9*).

The findings in this report are subject to at least six limitations. First, the sample of workers who participated in interviews might not be representative of poultry processing workers in Maryland or meat and poultry processing workers more broadly. Managers might have been biased in their selection of workers to participate, and workers who were out sick or otherwise absent at the time of the interviews were excluded. Also, the demographics of workers in Maryland might differ from populations in other parts of the United States. Second, the interviews were conducted in three languages, and some questions might have been misinterpreted as a result of translation. Third, much of the information was obtained by self-report, which could be subject to social desirability bias. Fourth, employment records combined race and ethnicity into a single variable and might have underestimated the Hispanic/Latino population. Fifth, although many workers at the poultry plants were tested for SARS-CoV-2, testing results could not be linked with the survey data, so it was not possible to calculate the actual risk for confirmed disease associated with each factor. Finally, interviews were conducted in May, when movement and community activities in Maryland were limited by closures and restrictions; the frequency of activities outside the home might have increased in the weeks after the interviews.

This investigation suggests that foreign-born and U.S.-born workers in poultry processing facilities likely face some different risk factors for SARS-CoV-2 transmission, and these factors might vary inside and outside the plant. Collecting data that include country of birth can therefore be used to inform public health practice (*10*). Though many prevention measures will benefit all workers, employers and health departments might consider placing special emphasis on the risk factors facing vulnerable groups, including foreign-born workers. For example, in the workplace, engineering and administrative controls can be tailored to the production floor. In community settings, information can be disseminated about how to more safely navigate common situations including carpools and close living quarters, and regulations such as mask mandates can also be considered. In addition, communities can increase sustained awareness and adherence to COVID-19 mitigation and prevention measures and guidance by collaborating with community-based organizations, such as labor groups and religious congregations that are directly led by persons from affected populations. These community-based organizations are well-positioned to disseminate culturally and linguistically tailored messages to foreign-born workers and the wider community.

**Summary**

**What is already known about this topic?**

Workers at meat and poultry processing facilities are at increased risk for SARS-CoV-2 infection and are disproportionately foreign-born.

**What is added by this report?**

Compared with U.S.-born poultry workers, foreign-born workers at two Maryland facilities had higher odds of working on the production floor and of living with other poultry workers and lower odds of participating in social gatherings and visiting businesses during the preceding week.

**What are the implications for public health practice?**

Engineering and administrative controls might reduce SARS-CoV-2 transmission risk for workers on the production floor, many of whom are foreign-born. Culturally and linguistically tailored messages should be disseminated about mitigation measures, particularly those pertaining to carpools and close living quarters.

## Footnotes

* According to the Migration Policy Institute, immigrants represent 17% of all civilian-employed workers in the United States and 37% of meat processing industry workers. https://www.migrationpolicy.org/content/essential-role-immigrants-us-food-supply-chain.

† At this time, no reports suggest that COVID-19 can be transmitted to humans by food or food packaging. https://www.usda.gov/coronavirus/food-supply-chain#food-safety.

§ https://www.cdc.gov/coronavirus/2019-ncov/downloads/community/organizations/carpooling-fs.pdf.

¶ https://www.cdc.gov/coronavirus/2019-ncov/daily-life-coping/living-in-close-quarters.html.

** 45 C.F.R. part 46, 21 C.F.R. part 56; 42 U.S.C. Sect. 241(d); 5 U.S.C. Sect. 552a; 44 U.S.C. Sect. 3501 et seq.

†† Data on mask usage was missing for one shared commuter, so the percentage who wore masks was calculated using a denominator of 127 workers.

§§ “Assigned multiple areas” refers to jobs that involve regular movement around all areas of the facility or that avoid the production floor altogether (e.g., maintenance or quality assurance).

¶¶ The studies cited did not statistically control for nonwork factors, although genotyping provided evidence that the initial outbreak in Germany was primarily caused by transmission on the processing floor, rather than in shared living quarters or carpools. Shared living quarters and carpools were likely confounding factors in a second outbreak at the same facility in Germany.
